# A Systematic Review of Data-Driven Attack Detection Trends in IoT

**DOI:** 10.3390/s23167191

**Published:** 2023-08-15

**Authors:** Safwana Haque, Fadi El-Moussa, Nikos Komninos, Rajarajan Muttukrishnan

**Affiliations:** 1 Department of Electrical and Electronic Engineering, School of Science & Technology, City, University of London, Northampton Square, London EC1V 0HB, UK; safwana.haque@city.ac.uk (S.H.); nikos.komninos.1@city.ac.uk (N.K.); 2 BT Group PLC, Ipswich IP5 3RE, UK; fadiali.el-moussa@bt.com

**Keywords:** IoT, datasets, machine learning, cyberattack, intrusion detection, threat detection

## Abstract

The Internet of Things is perhaps a concept that the world cannot be imagined without today, having become intertwined in our everyday lives in the domestic, corporate and industrial spheres. However, irrespective of the convenience, ease and connectivity provided by the Internet of Things, the security issues and attacks faced by this technological framework are equally alarming and undeniable. In order to address these various security issues, researchers race against evolving technology, trends and attacker expertise. Though much work has been carried out on network security to date, it is still seen to be lagging in the field of Internet of Things networks. This study surveys the latest trends used in security measures for threat detection, primarily focusing on the machine learning and deep learning techniques applied to Internet of Things datasets. It aims to provide an overview of the IoT datasets available today, trends in machine learning and deep learning usage, and the efficiencies of these algorithms on a variety of relevant datasets. The results of this comprehensive survey can serve as a guide and resource for identifying the various datasets, experiments carried out and future research directions in this field.

## 1. Introduction

Technology is a rapidly evolving paradigm that is especially difficult to keep up with in the field of computing. This can be mainly accredited to the advancements made in semiconductor chips, which are continuously improved and exploited for research purposes. Some of the most recent buzz terms that can be commonly heard and are of relevance to this paper are machine learning (ML), federated learning (FL), blockchain and Internet of Things (IoT). These technologies can be further combined with one another to improve their individual outputs or efficiency and to generate an alternate byproduct or result. For example, FL can be used to ensure or enhance data privacy in the IoT and ML can be used to make automated predictions in IoT devices. On the other hand, blockchain can be used to improve trust and transparency in data transactions in IoT networks.

IoT, which is the focus of this paper, is a term coined by Kevin Ashton in 1999 [1] but only gained traction in 2013. Since 2017, IoT has grown tremendously and will continue to do so at an even greater rate according to market and industry surveys [2,3,4,5,6]. IoT has penetrated every sector of life, encompassing transportation, health, communication, agriculture, homes, etc., with even traditional devices having become ‘smart’, e.g., smart locks, smart cars, smart fridges, smart lights, smart speakers and smart watches. According to [7], as of 2020, there was an equal number of IoT and non-IoT devices in the world, and the amount of the former is estimated to triple by 2025. While making life easier, this explosive growth has introduced many related concerns, such as the need for more speed, storage, capabilities, efficiency, etc., which researchers are continually trying to address and improve.

One of the biggest growing concerns, however, is the security and privacy of users, data, devices and the IoT network, which are often overlooked by both manufacturers and consumers. Implementing failsafe systems can be a painstaking process, yet the failure to do so can lead to serious repercussions for both individual users and companies. Cybercrimes are very common and already impact existing home IoT networks. A recent incident reported by the British Broadcasting Corporation (BBC), for instance, revealed how a family became suspects to a cybercrime that involved child abuse, to the detriment of their domestic life, income and mental health, the crime most likely having occurred via the hacking of their Wireless Fidelity (Wi-Fi) router, whose default password settings had not been changed [8]. Most cyberattacks commonly result from exploiting security vulnerabilities, such as weak/default password usage, poor update management, insecure interfaces, lack of user and data privacy, poor user awareness, lack of vendor standardization and many more.

Numerous steps must be continually taken to ensure that cybersecurity is maintained. These include the raising of user awareness/cyber education, security policy implementations, security software and tools (such as antivirus, firewalls, etc.) and, more recently, automated measures using machine and deep learning (DL) techniques. Exhaustive research has been carried out for conventional network and data security, but such work is severely lacking in emerging fields such as IoT. For example, numerous datasets have been generated and created by various studies and researchers on general-purpose networks, the earliest of which—known as the DARPA (Defense Advanced Research Projects Agency) dataset—dates back to 1998 [9]. Other datasets, found in [10,11,12], have been used to design intrusion detection and prevention systems (IDSs and IPSs, respectively). With respect to those widely used to train ML algorithms for IoT networks, older datasets, such as Knowledge Discovery in Databases (KDD) and Network Security Laboratory Knowledge Discovery in Databases (NSL-KDD), are believed to have shortcomings, e.g., there are a large number of duplicate records that could skew the machine training and learning process in the KDD dataset [13], and NSL-KDD, though an improvement over KDD, does not include more recent attack classes and IoT network properties. UNSW-NB15 [14] (by the University of New South Wales) and CIC-IDS2017 and CIC-IDS2018 [15] (by the Canadian Institute for Cybersecurity) are the more recent datasets used for IoT ML training, but as these datasets are not primarily concerned with IoT networks attack detection becomes limited.

IoT-specific datasets for the purpose of anomaly and attack detection have only been created and studied by researchers in the last few years, with significant results emerging from 2018. The objectives of this paper are primarily to:

- Highlight IoT-specific datasets: identify and emphasize datasets specifically designed or curated for IoT networks and applications and mainly for attack detection. These datasets contain information from IoT devices and sensors captured during various types of cyberattacks that are crucial for algorithm development and the evaluation of attack detection in the IoT domain.
- Compare IoT-specific datasets: compare different IoT datasets tailored for attack detection, considering their characteristics, attack scenarios and the diversity of attack types represented. Typical datasets may include simulated attacks, real-world attack traces and data from controlled IoT testbeds.
- Explore the ML and DL techniques carried out on them: investigate the application of ML and DL techniques to IoT-specific attack detection datasets, which may involve anomaly detection, behavior analysis, pattern recognition and classification methods to identify and mitigate various attacks targeting IoT systems.
- Observe any other future trends of study: examine emerging trends in IoT attack detection research and datasets.
- Outline research gaps for future studies or improvements: identify research gaps and propose improvements related to IoT attack detection datasets and methodologies. Potential areas of focus may include more realistic attack simulations, diverse data sources, addressing class imbalance in attack data and investigating the use of federated learning for distributed attack detection in IoT environments.

## 2. Background Study

### IoT Architecture and Threat Mapping

**Figure 1:**
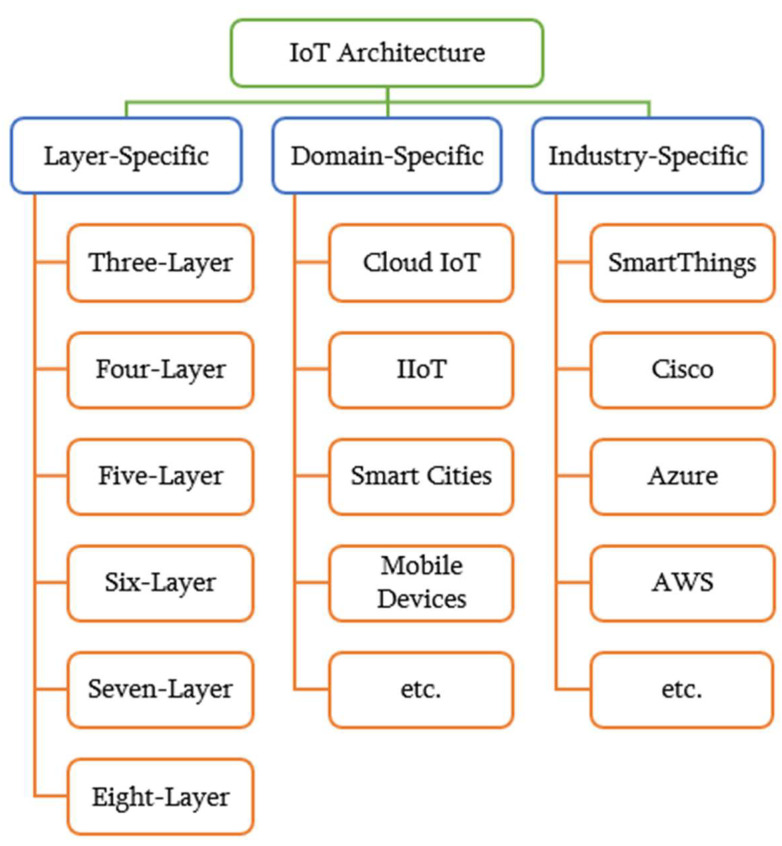
IoT architecture classifications.

Before delving into the solutions to IoT threats, attacks and problems, it is important to look at the various layers of the IoT architecture and the threats that are peculiar to them. This can be a daunting task as there are numerous ways that architectures have been classified according to layers [16,17] and domains [18,19,20], while some classifications are specific to the industries in which such architectures are used [21,22,23], as shown in Figure 1.

Layer-specific architectures can feature between three and eight layers. In fact, there could be multiple definitions and design specifications for an n-layer architecture. For instance, [24,25,26] have all defined five-layer architectures differently; the differences concern functionalities, the problems tackled, the technology considered or the methodology used. Similarly, three-layer or four-layer architectures also have multiple versions. These three architectures—of between three and five layers—are also found to be the most commonly classified and referenced model types [21,27,28,29]. Domain-specific architectures focus on a particular sector, e.g., cloud [18], industrial IoT (IIoT) [19], mobile devices [30], smart cities [20], etc. Industry-specific architectures are those customized by companies or businesses to suit their needs and services, e.g., the SmartThings architecture [31], the Cisco reference model [32], the Azure IoT reference architecture [33], the AWS (Amazon Web Services) IoT architecture [34], Google IoT Cloud Core [35], etc.

It can be seen from these architectures that they all differ in their outlooks, designs, functionalities and limitations. Furthermore, as IoT networks are highly distributed (spanning numerous networks and large coverage areas) and heterogenous (comprising different components and technologies), often operating in real time with cloud or big data, they need to be scalable to enable the growth of the networks and the integration of different components without affecting the quality of service, while also complying with the required set standards and, at the same time, ensuring the privacy and security of data and users involved in the communication process. All these factors make it difficult for researchers to come up with a single standardized architecture that encompasses all the requirements and functionalities that can be implemented without hampering the network’s performance. Despite all their differences, however, most architectures have the basic elements of an IoT system, which are: the **things** that constitute the physical devices of the network, such as the IoT sensors; the **network infrastructure**, such as the routers; the **cloud infrastructure** responsible for data management, storage and computation; and the **application or software end** that provides the human–computer interaction. These are usually categorized as a physical/perception/sensing layer, a network/transport layer, a middleware layer and an application layer, respectively.

Previous studies have largely tried to map threats to these three (physical, network and application) or four (physical, network, middleware and application) layers. For instance, the studies [36,37,38,39,40] show different types of IoT attacks, categorization, effects of the attacks and the existing countermeasures to help mitigate them. On the other hand, [41] mapped various attacks according to the security features affected, i.e., confidentiality, integrity, availability, accountability, auditability, privacy, trustworthiness and non-repudiation. Different layers have different characteristics and functions which are carried out and regulated by different protocols. The authors of [42,43] outlined the vulnerabilities, attacks and countermeasures of protocols found in the different layers, and [44] categorized emerging threats that could result from exploiting IoT device features based on mobility, interdependence, diversity, myriad, ubiquity, constrained, diversity, intimacy and unattended. The most efficient traditional and existing solutions implemented in IoT networks today are perhaps encryption and cryptographic solutions, until they are cracked and become outdated. Every layer can be secured with these means, e.g., Advanced Encryption Standard (AES) in the physical layer, Secure Sockets Layer (SSL) and Transport-Layer Security (TLS) in the application layer, etc. [42,45]. However, as [46] demonstrated, the AES-CCM (Advanced Encryption Standard Counter with Cipher Block Chaining Message Authentication Code) encryption mechanism within Philips Hue smart lights can be exploited by a single malicious bulb to cause a cascading effect in a city that can result in a power outage, for example, or other nefarious outcomes.

In addition to these traditional techniques, researchers have tried to explore more dynamic solutions, such as ML, DL and FL, as routes to enhancing IoT network security. However, to implement these techniques, datasets are needed to test and train against certain attacks in a network, and, as stated earlier, until recently, datasets for general-purpose networks were used to test and train IoT networks. As captured in Table 1, datasets for IoT networks are still few in number. The remainder of this study looks at the existing IoT datasets, their key characteristics, the ML and DL techniques applied to these datasets and future prospects.

## 3. Research Methodology

The research methodology used in carrying out this systematic review followed the guidelines set by the Preferred Reporting Items for Systematic Reviews and Meta-Analyses (PRISMA) [47]. The steps involved in the study are as follows:

- Defining the research questions (RQs) the review addresses.
- Outlining the information sources used to retrieve relevant information.
- Determining the keywords used to perform search queries in the databases.
- Filtering of information based on inclusion and exclusion criteria.
- Representation of the results found in relation to the research questions defined.

### 3.1. Research Questions

This study looked into IoT-related datasets and the automatic attack detection techniques used in relation to them. The research questions formulated for the purpose of this survey were:

- RQ1: What are the datasets created specifically for the study of IoT networks and their security?
- RQ2: Are there any similarities or differences among these datasets?
- RQ3: What ML and DL techniques have been applied to these datasets for attack detection?
- RQ4: Are any other methods applied to these datasets for attack detection?

### 3.2. Information Sources

Electronic searches to retrieve relevant papers related to this study were conducted on various databases. The databases selected have a large collection of articles and have a high reputation in the scientific world. The databases used and their respective access links are as follows:

- ACM (Association for Computing Machinery) digital library (https://dl.acm.org);
- Elsevier (https://www.elsevier.com);
- Google Scholar (https://scholar.google.com);
- IEEE Xplore (Institute of Electrical and Electronics Engineers Explore) (https://www.ieee.org);
- ScienceDirect (https://www.sciencedirect.com);
- SpringerLink (https://link.springer.com).

### 3.3. Keyword Search Queries

Search queries were formulated using various keywords to find answers relevant to the research questions outlined above. Keywords were combined with Boolean operators to form appropriate search queries to retrieve relevant answers to the research questions. The search queries used are listed below and are numbered according to the research questions answered for easier understanding.

- RQ1: (IoT OR Internet of Things) AND (Datasets) OR (Attack Detection OR Security);
- RQ2: (IoT OR Internet of Things) AND (Datasets) AND (Feature OR Feature Set);
- RQ3: (ML OR Machine Learning) OR (DL OR Deep Learning) AND (IoT OR Internet of Things) AND (Attack Detection);
- RQ4: (FL OR Federated Learning) OR (Solutions OR Countermeasures) AND (IoT OR Internet of Things) AND (Attack Detection OR Security).

### 3.4. Filtering Criteria

**Figure 2:**
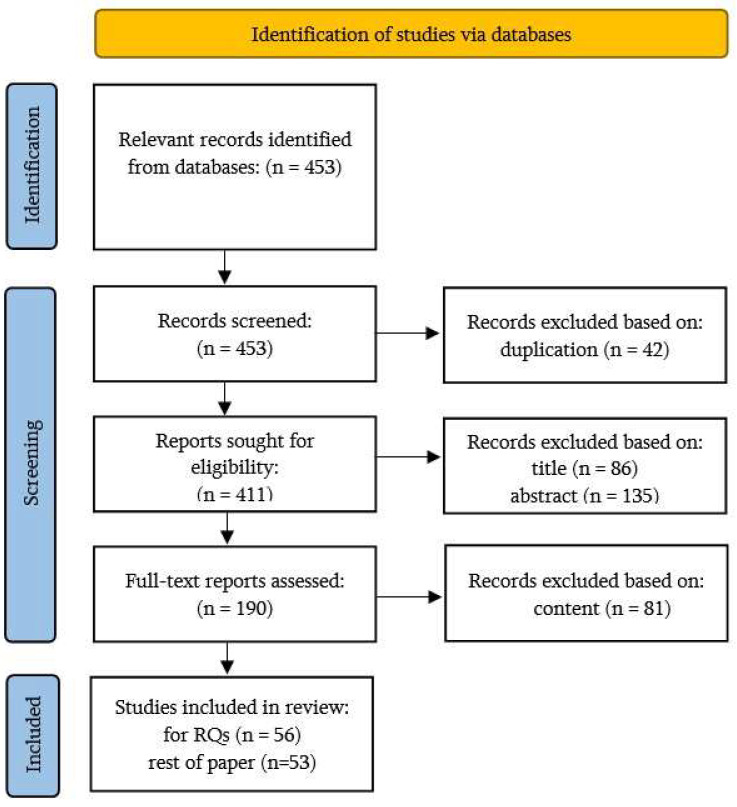
Selection process of articles based on inclusion and exclusion criteria.

Inclusion and exclusion filtering criteria were formed to retain the papers most relevant to this survey. The overall screening, elimination and selection process of articles is illustrated in Figure 2.

Inclusion criteria:

- Strict focus on IoT datasets: This study considers only research studies that exclusively pertain to Internet of Things (IoT) datasets. Any datasets or studies related to non-IoT datasets or general-purpose networks are deliberately excluded to maintain a clear and specific focus on IoT-specific data.
- Timeframe considered: The inclusion is limited to research articles published between January 2018 and May 2023. Articles published before 2018 or after May 2023 are not included so as to analyze recent developments and trends within a defined period.
- Source from reputable databases: Selected articles are sourced from reputable databases, such as academic journals and conference proceedings. Only articles that are already published or in the process of being published (in press) are considered for inclusion.

Exclusion criteria:

- General-purpose networks for IoT security: Research studies using general-purpose networks for IoT security analysis are excluded. The focus is solely on datasets and studies directly related to IoT networks and applications.
- Removal of duplicate articles: Any duplicate articles, whether they have the same content but different publication dates or are obtained from different search criteria or databases, are eliminated to avoid repetition and maintain data integrity.
- Articles not meeting the inclusion criteria: Articles that do not meet the specific inclusion criteria are excluded from the study. This ensures that only relevant and suitable studies related to IoT datasets are included in the analysis.

By applying these well-defined inclusion and exclusion criteria, the study aimed to gather a focused and relevant set of research articles directly related to IoT datasets. This approach ensured the accuracy and significance of the findings, providing valuable insights into IoT-specific data and their applications.

## 4. Survey Findings

### 4.1. RQ1: What Are the Datasets Created Specifically for the Study of IoT Networks and Their Security?

The survey addresses this research question by finding datasets that have been created using IoT devices in either a simulated environment or a physical network. In most cases, the IoT networks created are exposed to attacks and the network behavior is studied and analyzed under various attack conditions. Benign and attack data are collected and used to train ML and DL algorithms to create intrusion detection systems (IDSs). Ten datasets were found that are being studied and experimented on as part of this survey. Brief descriptions of these datasets are given below, while details of their attack capabilities can be found in Table 1.

1. **Bot-IoT** [48] is a simulated dataset created to study and analyze network forensics using ML and DL techniques. It is based on five IoT scenarios consisting of a weather station, a smart fridge, motion-activated lights, a remotely activated garage door and a smart thermostat. These simulated environments were exposed to three categories of attacks: information gathering (port scans, operating system (OS) fingerprinting); denial of service (Transmission Control Protocol (TCP), User Datagram Protocol (UDP), Hypertext Transfer Protocol (HTTP) for both denial of service (DoS) and distributed denial of service (DDoS)), and information theft (keylogging and data theft), which are commonly exploited by botnets (bots). This dataset consists of more than 72 million packet capture (PCAP) records. The distribution of attack records is not uniform, however, with the information theft attacks having the least number of records.
2. **IoT Network Intrusion Dataset** [49] **(IoTNID)** was created using two real devices: a camera and a speaker. The dataset consists of reconnaissance, man-in-the-middle (MiTM), DoS and Mirai attacks. All the attack packets except those of Mirai were captured using the Nmap tool, while the Mirai attack packets were generated using a laptop.
3. **IoT-23** [50] is a dataset created using three physical IoT devices: a Philips HUE smart Light Emitting Diode (LED) light, an Amazon Echo device and a Somfy smart door lock. These devices were set up to model 20 different malware scenarios and 3 benign scenarios (one for each device). Each malware scenario was exposed to a botnet (bot) attack, such as Mirai, Gafgyt, Torii, etc. This dataset was manually analyzed to provide benign and attack traffic features.
4. **MedBIoT** [51] is a dataset that tries to emulate a medium-sized network consisting of 80 simulated devices and 3 real devices. The devices used were a switch, a light bulb, a lock and a fan. The setup was exposed to three types of botnets: Mirai, BASHLITE and Torii. This dataset aims to provide data for intrusion detection of botnets.
5. **MQTT-IoT** [52] is a dataset based on a publish/subscribe message protocol called Message Queue Telemetry Transport (MQTT) used in the application/middleware layer. It is based on a simulated setup comprising 12 IoT sensors in four different attack scenarios (Table 1) and one benign scenario. This dataset was intended to be used for intrusion detection using ML techniques.
6. **MQTTset** [53] is another dataset based on the MQTT communication protocol, in this case aimed at aiding the application of ML techniques in MQTT networks. The setup was simulated using eight different sensors of the following types: temperature, light, humidity, carbon monoxide (CO) gas, motion, smoke, door and fan to exploit five MQTT network attacks. This dataset removes features such as source and destination IP (Internet Protocol) addresses, port addresses and communication times among others that can be found in other datasets and focuses mainly on MQTT-based features.
7. **N-BaIoT** [54]**:** The Network-based Detection of IoT (N-BaIoT) dataset was created using nine IoT devices, namely, two doorbells, one thermostat, one baby monitor, four security cameras and one webcam. These devices were of different makes and models. The network setup was exposed to two types of botnet attacks: Mirai and BASHLITE. Each of these botnets has other attacks, as specified in Table 1. This dataset comprises both benign and attack traffic intended for the study and detection of botnet attacks.
8. **ToN_IoT** [55] is a dataset that aims at addressing the properties of both IoT and IIoT by collecting data from telemetric sources, operating systems and network data, hence the name ToN_IoT. Nine types of attacks were studied on the seven types of sensors specified in Table 1. This dataset explores the interaction of network elements across the edge, fog and cloud layers and tries to provide data for intrusion detection in large-scale IoT network scenarios.
9. **Edge-IIoTset** [56]**:** This is another dataset that was created to study IoT and IIoT devices and networks. Its design architecture consists of seven layers and 12 IoT (e.g., sound detection sensor, ultrasonic sensor, etc.) and IIoT devices (servo motor, stepper motor, etc.) The testbed was tested with 15 attacks which were categorized into 5 broad attack categories.
10. **CICIoT2023** [57] is an IoT-based dataset that is the largest (as of 2023) in terms of the number of devices used to set up the network topology and the number of attacks studied. A total of 105 devices were used to design the testbed, and 33 attacks were carried out on the network for data collection, which were broadly classified into 7 attack categories. These attacks were carried out on the IoT devices using other IoT devices. This dataset also included Zigbee and Z-wave devices along with other IoT devices.

**Table 1:** IoT datasets summary.

	Year	Testbed Setup	Device Used	Attacks	Normal Traffic Gen Tool	Attack Traffic Gen Tool	Network Sim Tool	Packet Capture Tool
**Bot-IoT** [58]	2018	Virtual	5 devices simulated: smart refrigerator, smart garage door, weather monitoring system, smart lights, smart thermostat	Information gathering (service and OS scanning), denial of service (TCP, UDP, HTTP DoS and TCP, UDP, HTTP DDoS), information theft (keylogging, data theft)	Ostinato software [59]	Hping3 [60], Nmap [61], xprobe2 [62], golden-eye [63], Metasploit [64]	Node-red [65]	Tshark [66]; features extracted with Argus [67]
**N-BaIoT** [68]	2018	Real	9 real devices of types: doorbell, thermostat, baby monitor, security camera, webcam	BASHLITE (scan, junk, UDP flooding, TCP flooding, COMBO attack) and Mirai (scan, ack flooding, syn flooding, UDP flooding, UDP plain flooding)	N/A	Binaries and source code of BASHLITE and Mirai, respectively	N/A	Wireshark [69]
**IoTNID** [49]	2019	Real	2 real devices: Wi-Fi camera, speaker	Scanning (host, port, OS), man-in-the-middle, DoS attacks, Mirai (UDP, ACK, HTTP flooding, brute force)	N/A	Nmap	N/A	Monitor mode of wireless network adapter
**IoT-23** [70]	2020	Real	3 physical: speaker, light bulb, door lock	Mirai, Torii, Hide and Seek, Muhstik, Hakai, Internet Relay Chat Botnet (IRCBot), Hajime, Trojan, Kenjiro, Okiru, Gagfyt	N/A	Malware sample in a Raspberry Pi	N/A	Zeek [71]; features extracted with Zeek
**MedBIoT** [72]	2020	Mixed	80 virtual, 3 physical: switch, light bulb, lock, fan	Botnet malware: Mirai, BASHLITE and Torii	Scripts to trigger actions	Mirai and BashLite source codes, Torii sample	Docker [73]	tcpdump [74]; features extracted with Splunk [75]
**MQTT-IoT** [76]	2020	Virtual	12 MQTT sensors simulated	Aggressive scan, UDP scan, Sparta Secure Shell (SSH) brute force, MQTT brute-force attack	“Publish” MQTT command	Nmap, MQTT-PWN [77]	Virtual machines, VLC [78]	tcpdump
**MQTT set** [79]	2020	Virtual	10 simulated devices: temperature, light intensity, humidity, CO gas, motion, smoke, door opening/closure and fan status	Flooding denial of service, MQTT Publish flood, Slow DoS against Internet of Things Environments (SlowITe), malformed data, brute-force authentication	IoT-Flock [80]	MQTT-malaria [81], IoT-Flock, Message Queuing Telemetry Transport Security Assistant (MQTTSA) [82]	IoT-Flock	Eclipse Mosquitto [83]
**ToN_ IoT** [84]	2020	Mixed	7 simulated sensors: fridge, garage door, GPS tracker, modbus, motion light, thermostat, weather sensor	Scanning, DoS, DDoS, ransomware, backdoor, injection, cross-site scripting, password and man-in-the-middle attacks	JavaScript in Node-RED	Nmap, Nessus [85], Python script, Metasploitable3, bash scripts on DVWA [86] and Security Shepherd [87], CeWL (Custom Word List generator) [88], Hydra [89], Ettercap tool [90]	NSX-VMware [91], Node-RED	Data logger on Node-RED server, Zeek
**Edge-IIoT** [92]	2022	Real	12 physical IoT and IIoT devices	DoS/DDoS (TCP SYN, UDP, HTTP, ICMP), information gathering (port scan, OS fingerprinting, vulnerability scan), MiTM (DNS and ARP spoofing), injection attack (XSS, SQL injection, uploading attack), malware (backdoor, password cracking, ransomware)	N/A	Hping3, Slowhttptest [93], Nmap, Netcat [94], Xprobe2, Nikto [95], Ettercap, XSSer [96], SQLmap [97], CeWL, OpenSSL cryptography toolkit [98]	N/A	Wireshark, Zeek and Tshark for feature extraction
**CICIoT 23** [99]	2023	Real	67 IoT devices, 38 Zigbee and Z-wave devices	33 attacks in 7 categories (DDoS, DoS, Recon, web-based, brute force, spoofing, Mirai)	N/A	Hping3, udp-flood, slowloris, golang-httpflood, nmap, fping [100], DVWA, remot3d [101], BeEF [102], hydra, Ettercap, Mirai code	N/A	Wireshark, tcpdump and dpkt package for feature extraction

### 4.2. RQ2: Are There Any Similarities or Differences among These Datasets?

To address this research question, the IoT-related datasets found in the literature were compared. It was observed that all the datasets surveyed in this study vary in respect to the number and types of devices used in the setup; the type of setup, whether simulation, real or mixed; the attacks the devices were exposed to, etc., as shown in Table 1. However, there are similarities among them which are discussed below:

1. **Features:** Bot-IoT is the earliest IoT dataset considered in this study and has been utilized by a number of researchers to carry out ML techniques for intrusion detection training. Even though this dataset employs the MQTT protocol, similar to the MQTT-IoT and MQTTset datasets, its feature set has no MQTT-based features, such as those found in the latter two, which are the only datasets that contain MQTT-related features. From Table 2, which shows the features common among the datasets studied, it can be seen that N-BaIoT and MedBIoT have 100 similar features to each other but have no common features with other datasets. Similarly, MQTT-IoT and MQTTset have MQTT-related features that are not found in other datasets. Over 15 features common to the ToN_IoT and IoT-23 datasets were also seen.
  The most common features found amongst the datasets were the five-tuple network flow features (source/destination IP address, source/destination port and protocol) and timestamps. A difference in opinion and research carried out regarding these features has been observed. While some studies, such as [79], removed common features like the source/destination IP and port addresses, as well as communication times, from their MQTTset to allow the identification of features independent of a particular connection/communication, others, such as [103], used these features in the IoT Network Intrusion Dataset to carry out ML training and testing for attack detection. These features, while important in identifying a network flow, carrying out network configurations and troubleshooting, could skew the ML training processes, leading to overfitting and the generation of high prediction rates. Other features, such as sequence or identification numbers, found in IoT-23, Bot-IoT, Edge-IIoT and IoTNID, could have similar effects.
  Most datasets have one or more of the three features (attack, category and subcategory labels) that are used to tag a flow as benign, attack or type of attack. The attack label is used to tag a traffic flow as either benign or attack traffic, which are sometimes denoted as 0 and 1, respectively. On the other hand, the category and subcategory labels are used in datasets where there are a number of different attack types and classes, e.g., the category is used to indicate that a flow belongs to a DoS attack while the subcategory indicates if it was a UDP, TCP, HTTP or ICMP (Internet Control Message Protocol) DoS attack. These features are not used in the training process, however, but to measure the performance of ML models. The category and subcategory labels are useful for supervised learning where the model is trained for the detection of the related attack class, while the label is useful for both supervised and unsupervised learning. In datasets where the labels are not explicitly given, such as in N-BaIoT, MQTTset, etc., the PCAP or comma-separated values (CSV) files are collected and organized separately for each type of attack or normal class for easy identification.
2. **Attacks:** This is another important characteristic of an IoT dataset, as this would determine the type of attack an IDS would be able to detect when trained with the particular dataset. Table 3 shows the types of attacks carried out in the test environment to create the datasets. The attacks have been categorized to show the layer of architecture they belong to. As IoT networks do not have a standardized architecture yet, such as the Open Systems Interconnection (OSI) model used in a conventional network, the attacks have been mapped to the OSI model depending on the layer the attack exploits.
  For example, an application-layer attack targets the highest layer of the OSI model, exploiting the application-level protocols and services. Some of the attacks seen in this category were cross-site scripting (XSS), SQL injection and HTTP DoS attacks. The most common form of transport-layer attacks seen in these datasets were the TCP and UDP DDoS/DoS attacks which exploit the weaknesses of transport-layer protocols to overwhelm the network resources. Other layered attacks, such as ICMP flood/DoS attacks in the network layer, were observed, while only ARP (Address Resolution Protocol) spoofing was seen in the datalink layer. No physical layers have been studied in these datasets. Other malware or botnet attacks are more difficult to classify as they can span multiple layers.
  Some datasets, such as N-BaIoT, IoT-23 and MedBIoT, contained traffic related to botnet attacks only. The IoT_23 dataset contains the highest number of different botnets, while Mirai and BASHLITE are the most common types seen across all the datasets. DoS and reconnaissance attacks are the next most common attacks found in these datasets. Attacks related to IoT protocols, such as MQTT attacks, were contained only in the MQTT-IoT and MQTTset datasets. Attacks related to other IoT protocols, such as Constrained Application Protocol (CoAP) attacks, have not been explored. It was seen that as more datasets are created, the complexity in terms of the number devices or attacks explored increases. CICIDS23, which is the most recent IoT dataset in this study has the highest number of attacks and devices explored.
3. **Devices Used:** Table 1 shows the types of devices used in the experimental setups of the different datasets. It has been observed that there is a huge difference in the number and types of devices chosen for each type of dataset, ranging from just 2 devices in IoTNID to 105 devices in CICIDS23. MedBIoT uses 83 devices in its setup, of which 80 are virtual devices and 3 are physical devices. The MQTT-IoT dataset simulates 12 MQTT sensors to study the MQTT features and attacks, while CICIoT23 incorporates ZigBee and Z-wave devices in its setup. ToN_IoT and Edge-IIoT have included the modbus protocol and motor sensors to allow these datasets to be used for IIoT studies.

### 4.3. RQ3: What ML and DL Techniques Have Been Applied to These Datasets for Attack Detection?

These IoT datasets have been created to facilitate the study of the behavior of network parameters under different attacks and to devise means of either detecting or preventing attacks from occurring in a network. Table 4 shows the studies that have been undertaken by researchers to explore the performances of different ML and DL techniques on the available IoT datasets as either network intrusion detection or anomaly detection solutions. Any IDS designed with these datasets will be signature-based, meaning the IDS will be able to match the characteristics of a network flow with the attack flow it is trained with. An anomaly detection solution, on the other hand, will be trained to detect any traffic that deviates from the norm and alert the system. This has an added advantage in the sense that attack traffic may be easily identifiable. However, it is unable to identify the type of attack, which an IDS may be able to do.

It can be seen from Table 4 that newer ML techniques, such as DL, are gaining prominence. The advantage of DL algorithms, in comparison to ML algorithms, is that their performances can be improved by modifying their underlying hyperparameters. However, they can take longer [104] and have more processing overhead to train and test the model than their counter-ML algorithms. For these reasons, researchers have adopted a similar approach to DL as they have with ML, which is selecting the minimum and best features of a dataset to train an algorithm, as shown in Table 5. It can be seen in [105], among other studies, that the runtime is reduced with a smaller feature set without (significantly) affecting the efficiency of the algorithm.

Some scientists, on the other hand, have tried to combine algorithms or create different ones similar to ensemble techniques [103,106]. Overall, it was seen from [79,105] and [107], for example, that tree-based algorithms, such as random trees (RTs), random forests (RFs), etc., performed better on average compared to others. Algorithms like Naïve Bayes (NB), though faster, had poorer performance comparatively [84,108,109]. It was also observed that the most commonly used ML algorithms were tree-based, while neural networks (NNs) are the most common for DL algorithms. These results can be seen in Table 4. Furthermore, Table 4 shows the accuracies of different algorithms according to different researchers. However, some studies did not present the results as accuracy values but incorporated other performance metrics, such as F-scores (or F1-scores).

**Table 4:** ML and DL techniques used on IoT datasets.

Dataset Used	Ref	Technique Used	Acc	ML	DL	Best-Performing Algorithm	Worst-Performing Algorithm
**Bot-IoT**	[105]	Adaptive Boosting (AdaBoost)	0.97			KNN	NB
		Iterative Dichotomiser 3 (ID3)	0.97				
		k-Nearest Neighbors (k-NN)	0.99				
		Multilayer Perceptron (MLP)	0.84				
		NB	0.79				
		Quadratic Discriminant Analysis (QDA)	0.87				
		RF	0.97				
	[109]	Bayes Networks	0.996			RT	NB
		C4.5 (Decision Tree-Based Classifier)	0.9999				
		NB	0.7341				
		PART (Partial Decision Tree)	0.9999				
		RF	0.9999				
		RT	0.9999				
		REPT (Reduced Error Pruning Tree)	0.9999				
	[10]	Convolutional Neural Network (CNN)	N/A			CNN	NB
		Deep Auto Encoder					
		Deep Belief Network (DBN)					
		Deep Boltzmann Machine (DBM)					
		Deep Neural Network (DNN)					
		Recurrent Neural Network (RNN)					
		Restricted Boltzmann Machine (RBM)					
	[58]	Long Short-Term Memory (LSTM)	0.9974			LSTM (worst time)	SVM (best time)
		RNN	0.9974				
		Support Vector Machine (SVM)	0.8837				
	[110]	Bayes Networks	0.9977			All performed extremely well and almost the same, but [110] considered NB to be best as it took the least time	
		C4.5	0.9999				
		NB	0.9979				
		RF	0.9999				
		RT	0.9999				
	[111]	RF	1			-	-
	[112]	Feedforward Neural Network	>0.99			-	-
	[113]	CNN	0.9602			All are in the same range but recall and F1-scores for data theft attacks are much lower than others	
		DNN	0.9576				
		RNN	0.9676				
**IoTNID**	[103]	Decision Tree (DT)	0.88			DT	LR and SVM
		Ensemble	0.87				
		Gaussian NB	0.73				
		Linear Discriminant Analysis (LDA)	0.70				
		Logistic Regression (LR)	0.40				
		RF	0.84				
		SVM	0.40				
**IoT-23**	[107]	Adaptive Boosting	0.87			RF	NB
		Artificial Neural Network (ANN)	0.66				
		NB	0.23				
		RF	1				
		SVM	0.67				
	[104]	DNN	0.984			Ensemble	RF
		LSTM	0.991				
		RF	0.893				
		Stacked or ensemble	0.997				
	[114]	Gradient Boost	0.9945			RF	MLP
		MLP	0.9942				
		RF	0.9986				
	[115]	k-NN	0.9994			RF	LR
		LR	0.9991				
		NB	0.9992				
		RF	1				
	[106]	RF	-			Own proposed method	AdaREPT
		REPT	-				
		Adaboost + REPT (AdaREPT)	-				
		Own proposed method	-				
	[116]	Adaboost	1			Almost all, but on further analysis by [116], DT and AdaBoost had the shortest times	NB
		DT	0.99				
		Extra Trees Classifier (ET)	1				
		k-NN	1				
		NB	0.99				
		RF	1				
**Med BIoT**	[72]	DT	0.9516			RF	k-NN
		k-NN	0.8706				
		RF	0.9766				
	[117]	DT	0.99			DT. 2-, 3- and 4-class classification performed using 7 features	k-NN, especially in terms of computational time
		ET	0.99				
		k-NN	0.89–0.97				
		RF	0.98–0.99				
**MQTT-IoT**	[76]	DT	0.9615			DT	SVM (Linear Kernel); average packet, unidirectional, bidirectional accuracies computed here
		Gaussian NB	0.8557				
		k-NN	0.8957				
		LR	0.9218				
		RF	0.8845				
		SVM (RBF Kernel)	0.9066				
		SVM (Linear Kernel)	0.8260				
**MQTTset**	[79]	DT	0.9779			All perform well; however, [79] shows that differences in results occur between balanced and unbalanced datasets, with NB scoring the least compared to others when a balanced dataset is used	
		Gradient Boost	0.9911				
		MLP	0.9468				
		NB	0.9879				
		RF	0.9942				
		Neural Network	0.9932				
	[113]	CNN	0.8977			All are in the same range but recall and F1-scores for brute force, malformed and flood attacks are much lower than for benign, DoS and SlowITe classes	
		DNN	0.9006				
		RNN	0.8929				
**N-BaIoT**	[118]	k-NN	0.9536			The authors of [79] combined different feature selection methods for comparison. RF performed better than k-NN in most cases	
		RF	0.9985				
	[68]	Autoencoders (AEs)	1			N/A	
	[119]	DT	>0.98			DT	k-NN
		k-NN	>0.94				
	[120]	CNN	N/A			CNN, DT and RF	RNN, LR
		DT					
		k-NN					
		LR					
		LSTM					
		NB					
		RF					
		RNN					
	[121]	MLP-ANN	N/A			N/A	N/A
	[117]	DT	0.98–0.99			DT. 2-, 3- and 9-class classification performed using 3 features	k-NN, especially in terms of computational time
		ET	0.99				
		k-NN	0.98–0.99				
		RF	0.98–0.99				
	[122]	LR	0.9998			LR	Proposed ANN
		Proposed ANN	0.964				
**ToN_ IoT**	[114]	Gradient Boost	0.94643			RF	Gradient Boost
		MLP	0.97842				
		RF	0.98075				
	[84]	Classification and Regression Trees (CARTs)	0.77			CARTs	NB
		k-NN	0.72				
		LDA	0.62				
		LR	0.61				
		LSTM	0.68				
		NB	0.54				
		RF	0.71				
		SVM	0.60				
	[111]	RF	0.9968 (for 8 features)			N/A	N/A
	[113]	CNN	0.9887			All are in the same range but recall and F1-scores for XSS are lower than other classes	
		DNN	0.9968				
		RNN	0.9998				
	[123]	AdaBoost	0.399			XGB	AdaBoost
		DT	0.934				
		k-NN	0.979				
		LR	0.777				
		NB	0.712				
		RF	0.937				
		SVM	0.780				
		Extreme Gradient Boosting (XGB)	0.983				
	[124]	Adaptive Boosting	0.5604			Ensemble stacking (used 3 of the best classifiers)	AdaBoost
		CatBoost	0.9934				
		DT	0.9917				
		ET	0.9936				
		Gradient Boosting (GB)	0.9766				
		k-NN	0.9459				
		RF	0.9875				
		XGB	0.9946				
		Ensemble Soft Voting	0.9947				
		Ensemble Stacking	0.9949				
	[125]	CNN	0.8847			Own	LSTM
		CNN+LSTM	0.8863				
		LSTM	0.8815				
		Own proposed method	0.9057				
**Edge-IIoT**	[92]	DNN	0.9467			DNN	DT
		DT	0.6711				
		k-NN	0.7918				
		RF	0.8083				
		SVM	0.7761				
	[125]	CNN	0.9495			Own	CNN + LSTM
		CNN + LSTM	0.87				
		LSTM	0.9445				
		Own proposed method	0.9496				
**CICIoT 2023**	[99]	Adaboost	0.6078			RF	Adaboost
		DNN	0.9861				
		LR	0.8023				
		Perceptron	0.8195				
		RF	0.9916				

Table 5 shows the various works that have been carried out using both the full feature set and the best feature set of the IoT datasets. Different selection methods have been explored, such as the use of ML/DL techniques by [105,115], deductive reasoning using various filtering criteria by [111,112] and statistical methods by [58,110,116,118]. Various numbers of best features have been selected by researchers, with some using as low as the best two, three and four features. The performance of ML and DL algorithms are most commonly evaluated using precision, accuracy, recall and F-measure metrics.

Despite various efforts, it was seen that some classes in the datasets did not yield promising results. For example, [107] found the prediction of benign traffic in IoT-23 to be poor, while [108] reported low precision rates for data theft and keylogging attack classes. Understanding the reasons behind these outcomes is important so that the datasets can be improved and newer ones without the same shortcomings can be generated in order to yield better detection results.

**Table 5:** Feature selection techniques explored on IoT datasets.

Dataset Used	Ref	Evaluation Methods Used	Number of Features Used	Best Feature Selection Technique
**Bot-IoT**	[105]	Precision, accuracy, recall, F-measure, processing time	Best 7, 13 and full set of features; results for accuracy, best and worst algorithms given for best 7 features	RF Regressor algorithm
	[109]	Accuracy, false-positive rate (FPR), precision, recall, time to build	Full set	N/A
	[10]	Accuracy, true-positive rate (TPR), FPR	Full set	N/A
	[58]	Accuracy, precision, recall, training time, FPR	Best 10 and full set	Correlation coefficient with joint entropy
	[110]	Accuracy, precision, recall, time to build model	Full set	Bijective soft method applied for ML selection
	[111]	Area under the curve (AUC), F-measure	Set of 4, 5, 6, 7 and 8 features	Deductive filtering
	[112]	Accuracy	29 features	Deductive filtering
**IoTNID**	[103]	Precision, recall, F-measure	83 features extracted from [49]	Shapiro–Wilk algorithm, correlation
**IoT-23**	[107]	Precision, recall, F-measure	Full set	-
	[104]	Precision, recall, F1-score, FPR, Matthews correlation coefficient (MCC), g-mean	Full set	N/A
	[114]	F1, AUC, mean square error (MSE), Gini	Full set	N/A
	[115]	Precision, recall, F1-score	Best 10	LR
	[106]	Precision, recall, F1-score	Full set	N/A
	[116]	Precision, recall, F1-score	Best 40% and full set; accuracy values given for entire feature set	Information gain, Gini impurity, correlation measure, Pearson’s correlation, consistency measure
**MedBIoT**	[72]	Precision, recall, F1-score	Full set	N/A
	[117]	Precision, recall, F1-score, accuracy, computational time, performance achieved	Multiple sets: 7 to 85	Pearson’s correlation, Fisher score, mutual information, Analysis of Variance (ANOVA), Recursive Feature Elimination (RFE), Sequential Forward Selection (SFS), Sequential Backward Selection (SBS)
**MQTT-IoT**	[76]	Precision, recall, F1-score	Full set	N/A
**MQTTset**	[79]	F1-score, training time, testing times	Full set	N/A
	[126]	Precision, recall, F1-score, accuracy	Full set	N/A
**N-BaIoT**	[118]	Accuracy, detection time	Best 4, 10, 18, 20 and full set	Fisher’s score, Pearson’s correlation coefficient, Sequential Forward Feature Selection, Sequential Backward Feature Elimination
	[68]	N/A	Full set	N/A
	[119]	F-score	Best 2, 3, 10; accuracy results for any set of features	Fisher’s score
	[120]	Precision, recall, F-score	Full set	N/A
	[121]	Precision, recall, F-score	Full set	N/A
	[117]	Precision, recall, F1-score, accuracy, computational time, performance achieved	3–68	Pearson’s correlation, Fisher’s score, mutual information, ANOVA, RFE, SFS, SBS
	[122]	Precision, recall, F1-score, accuracy, FPR, loss	19	LR
**ToN_ IoT**	[114]	F1, AUC, MSE, Gini	Full set	N/A
	[84]	Precision, recall, F-score	Full set	N/A
	[111]	AUC, F-measure	Set of 4, 5, 6, 7 and 8 features	Deductive filtering
	[123]	Recall, precision, F1-score, accuracy	20	Chi-square
	[124]	Recall, precision, F1-score, accuracy, MCC, AUC	22	Spearman rank correlation coefficient
**Edge-IIoT**	[92]	Recall, precision, F1-score, accuracy, learning rate, error, validation error, validation accuracy, training time, validation time for DL models	Full set only but showed 5 features important to each attack class	RF
**CICIoT 2023**	[99]	Accuracy, recall, precision, F1-score	Full set	N/A

### 4.4. RQ4: Any Other Methods Applied to These Datasets for Attack Detection?

It was observed that a different approach from the more traditional ML or DL is on the rise now. Known as federated learning, FL allows participating devices (in this case IoT devices or sensors) to retain their individual data (instead of sharing it with a server or datacenter) and to collaboratively train a shared prediction model. This method promotes privacy as node data are not exposed. Another advantage of this method is that data from devices can be non-IID (independent and identically distributed), meaning the devices could train the model at different times with different data sizes or parameters. This is a huge advantage, as IoT sensors differ in terms of their characteristics and the amount of information they gather.

An increasing number of studies using FL have been seen in the last two years. Seven of the discussed datasets in this study have been explored by researchers using FL, as shown in Table 6. It is more common to see the use of DL or neural networks (NNs) in FL than traditional ML algorithms. This can be accredited to the fact that DL and NN models are better at learning and computing complex patterns in data with the use of multiple layers and deep architectures. This also reduces the need for manual feature engineering, as DL and NN algorithms can automatically deduce important features in the data used. A key difference between FL and ML is the use and transfer of models instead of data between devices and the training/testing server that allows privacy preservation of data. This is made possible with the use of transfer learning, where DL models can be pre-trained and deployed on the IoT devices, thereby reducing the need to train models from scratch. However, despite these benefits, DL algorithms are more resource-consuming compared to ML algorithms, e.g., in terms of training time, memory consumption, computational time, etc., which would add to the overheads of IoT devices, as they are usually limited in resources.

It is important to devise means of achieving FL stability with a small number of epochs (local model iterations on the IoT device) and rounds (global model iterations between the IoT device and the server) to reduce the computational overhead on IoT devices and the network performance. It can be seen from Table 6 that some studies have up to 1000 rounds [127] and others up to 400 epochs [128]. Some others [129] deploy the training data to edge devices for local model training. However, though this reduces the burden on the IoT device, this approach could lead to data leakage through the sharing of data to a third party. A balance between deploying light FL models and achieving optimum performance is key to exploring these solutions for IoT attack detection.

**Table 6:** FL techniques used on IoT datasets.

Ref	Aggregation Method	No. of Clients	No. of Rounds	Metrics	ML/DL	Data	Dataset
[127]	FedAvg	4	1000	Accuracy, precision, recall, F1-score	CNN, DT, KNN, NN, RNN, RF, SVM	-	Bot-IoT
Implements Low-Complexity Cyberattack Detection in IoT Edge Computing (LocKedge) Shows complexity and CPU usage							
[92,113]	FedAvg	5, 10, 15	1, 50	Best client acc, worst client acc and global model acc	CNN, DNN, RNN	IID and non-IID	Bot-IoT, Edge-IIoT, MQTTset, ToN_IoT
Compares FL performances on Bot-IoT, Edge-IIoT, MQTTset and ToN_IoT datasets in terms of best/worst client and global model accuracies							
[129]	FedAvg	5 edge devices	8	Accuracy, precision, recall, F1-score	DNN	Non-IID	Bot-IoT, N-BaIoT
Compares centralized, distributed, localized and FL performances for zero-day attack Shows training time, latency, memory required for the above methodologies Shows the performances of five edge devices with the four methodologies used							
[130]	Mini-batch avg, multi-epoch avg	8	1–30	Accuracy, TNR, TPR, F1, threshold	MLP, AE	Non-IID	N-BaIoT
Studied the effects of all labels flipping attack, gradient factor attack, model cancelling attack using averaging, coordinate-wise median, coordinate-wise trimmed mean Showed computational and communication costs							
[131]	Multi-epoch aggregation	-	30	Loss value, accuracy	CNN, LSTM, Gated Recurrent Unit (GRU),	-	N-BaIoT
Shows loss value and training time of three MLs used for centralized and FL							
[132]	FedAvg	1–23 clusters acting as clients	50	Accuracy, precision, recall, F1-score, loss curve	-	Non-IID	IoT-23
Studies effect of clusters of trust between nodes and globally shared data							
[128]	Asyn DC Adam	1–5 nodes	400 Epochs	Accuracy, precision, recall, F1-score	Denoising AE (DAE)	Non-IID	IoT-23
Implements asynchronous FL using a delay compensated Adam (DC-Adam) approach Shows loss function convergence for training data							
[133]	FedAvg	-	10	Accuracy, precision, recall, F1-score	Ensemble with RF	-	MQTT-IoT
Multiview FL using bidirectional features, unidirectional and packet features							
[134]	FedAvg, Fed+	4, 10	1–300	Accuracy, recall, FPR, precision, F1-score	LR	-	ToN_IoT
Partitions data in basic (unbalanced), balanced and mixed scenarios using Shannon’s entropy							
[135]	FedAvg, FedProx, FedYogi	10	1–50	Accuracy, recall, FPR, precision,	DBN, DNN	non-IID	ToN_IoT
Explores the effect of data heterogeneity with different aggregation methods							

## 5. Future Research Directions

Developing and utilizing IoT-based datasets for IoT-related solutions is a step in the right direction, even though many IoT studies are still carried out on NSL-KDD, for example, a generic non-IoT dataset created in 1999. Researchers have struggled in the past to devise security measures for IoT networks using outdated datasets with deficiencies, such as a lack of modern attacks, imbalanced attack classes and absence of IoT devices. IoT networks are different from generic and conventional networks in a number of ways. For example, different device sensors in the same network could have different functional capabilities (complex, such as a TV, or simple, such as a door lock), different modes of working (e.g., continuous stream from a camera or intermittent status update from a light), etc.

With the release of new IoT-based datasets, it is hoped that they will help with the study of IoT networks and the devising of stronger security measures. However, the following considerations should be taken on board when designing and testing new IoT-specific datasets.

***Feature Identification, Selection and Extraction:*** It is important that researchers identify the unique features of IoT traffic that distinguish it from that of general-purpose networks. It is also important to understand if features that can uniquely identify a particular flow (e.g., source IP address, destination IP address, transmission time logs, etc.) are relevant and should be included in the feature set of a dataset, as stated in [79]. This raises the question that if, for instance, one of such features, such as IP address, has a high dependance value, then could that lead to some benign traffic with an IP of a malicious traffic flow being identified as malicious? Or, if an IP address that is recognized as benign traffic is used by an attacker, could that malicious traffic be wrongly classified as benign? The numbers of devices seen in these IoT datasets are generally small, except in MedBIoT and CICIDS2023. This could lead to misclassification if training models map certain IP addresses to certain attack classes when trained on these datasets and tested within different test environments. It is therefore essential to identify features that should be dropped for training in order to avoid overfitting or misleading results.

***Relationship between IoT Attacks and Architectural Layers:*** Similarly, it is important to explore and deduce relationships between the various attacks that occur in the different architectural layers (e.g., application, network and physical layers) of an IoT system and the distinguishing characteristics or features of these attacks. This knowledge would be useful in designing IDSs based on the dependent features for more accurate and targeted results. Also, it is important to understand the cascading effects and behaviors of interconnected IoT devices in a network under attack, if and how attacks migrate between layers, features that could be used to detect such attacks, if such features change as the attack progresses to a different layer and the subsequent damages that can be caused in such a network.

***Performance Evaluation and System Requirements:*** Different researchers have tried to reduce the training and testing times of datasets by selecting features of utmost importance without hampering the performances and efficiencies of the ML/DL algorithms used. Some of these deductions were made by using statistical methods, such as correlation coefficients, entropy, Fisher’s score, information gain, etc., as shown in Table 5. Others were made by manual deductions and reasoning, as seen in [79,111,112], while others, such as [105] and [115], used ML algorithms to select features. As these datasets are relatively new, they have not been fully explored yet. However, it is important to determine key features and the attacks they are related to so as to improve the efficiencies of IDS. Also, finding features that can interrelate and adapt with other datasets will be useful in developing scalable IDSs for real-world implementations. The performances of training models are usually seen to be evaluated in terms of accuracy, recall, precision, F-score, etc. It is equally important to understand the system requirements for implementing such a model for attack detection in terms of the memory required, time taken, energy consumed, etc., especially in an IoT network where hubs and devices have limited resources and capabilities.

***Standardization of Datasets***: It can be seen from the datasets studied in this paper that, though they differ in the various ways identified, botnets and DoS attacks are the more popular kind of attacks addressed in the datasets. This may be accredited to the fact that these attacks result in colossal damage when successful. However, there is a need to build standard and unified datasets that can be used to design IDSs with a wider attack set across different IoT platforms made up of a varied number of devices. The authors of [136,137,138] have tried to do this, where [136] combined non-IoT and IoT datasets, while [137,138] combined multiple IoT datasets. Additionally, it was further observed that when datasets with numerous attacks are created, the attack flows for the respective attacks are not evenly distributed, e.g., the number of flows for information theft in [58]; the XSS, fingerprinting, port scan and SQL injection attacks in [92] are much lower than other attack flows contained in the respective datasets. This led to insufficient training of the ML models and poor prediction results for the attacks seen in [58] and [92]. Similarly, a huge percentage of misclassification can be seen in [99], where attacks such as brute-force, reconnaissance, spoofing and web attacks have fewer data compared to the DoS/DDoS attacks, for example. Although, the overall predictions of attacks using these datasets are high, a class-by-class investigation of attack prediction shows low results for certain classes. It is therefore important to create datasets with an optimum number of data flows for each type of traffic classification to allow proper training and testing of ML/DL models.

***Exploring IoT Protocols and Technologies:*** In datasets such as MQTTset and MQTT-IoT based on MQTT, an application-layer protocol was seen. However, other application-layer protocols related to IoT, such as CoAP, XMPP (Extensible Messaging and Presence Protocol) or AMQP (Advanced Message Queuing Protocol), are still lacking today. It is also important to explore other IoT-related technologies, such as 6LoWPAN (IPv6 over Low-Power Wireless Personal Area Networks), BLE (Bluetooth Low Energy), Zigbee, Z-wave and NFC (near field communication), for example, to enable attack detection in networks using these technologies. It was observed that CICIDS2023 included Zigbee and Z-wave devices in its experimental setup along with other IoT devices. However, it is not clear how these devices differ in their behavior when infected or under attack, e.g., are there features that could be used to show that a Zigbee device is under attack and not a Z-wave device or vice versa? It is imperative to understand how or if devices or technologies are affected differently to one another when infected with attacks and, in turn, what unique features can be used to train ML or DL algorithms to detect attacks in networks with devices operating on different protocols, e.g., what MQTT or CoAP features are affected in an MQTT or CoAP setting, respectively, or are there network features that could show that a Bluetooth device is under attack in a network consisting of other types of devices, etc. It is therefore vital to not only have IoT datasets that are more complex in terms of size, number of devices, technology, attack detection capabilities and protocols in the future, but to also understand how these differences can be identified and used to design better and more efficient IDSs.

***Privacy and Federated Learning in IoT Security:*** Privacy is a security element that is often overlooked and even less understood by consumers. It is essential to start focusing on this area with the latest trend of FL as it allows the data of individual devices and communication to remain private and secure during the training and testing process. Table 6 shows some of the studies that have explored this technique using existing IoT datasets. Others, such as [139], have designed their IoT networks with IoT sensors for anomaly detection using FL. It was seen from Table 6 in this review that DL techniques are applied in FL attack detection, which are more resource-intensive and computationally demanding compared to ML techniques. Table 4 shows that using ML for attack detection gave high accuracies in numerous studies. It is therefore important for researchers to justify the use and implementation of DL in IoT networks where resources are constrained and to develop lighter means for such deployments. It is also essential to understand how FL in IoT networks consisting of varied numbers and types of devices can be implemented efficiently, e.g., identifying how a simple device (e.g., a smart bulb/switch) and a more complex device (e.g., a smart camera/TV) participate in an FL setting, when and how the FL training takes place in such devices according to their capabilities without affecting the performance of the device or network, and the features important to the type of device and also the attack for efficient FL training and attack detection. In addition to the usual attacks that devices and networks are affected by, FL is vulnerable to additional attacks, such as poisoning attacks, model inversion attacks, Byzantine attacks, etc. It is therefore essential to devise means of deploying FL in a robust and feasible manner while moving forward in this field.

## 6. Conclusions

This review has provided a foundation for understanding the current state and potential trajectory of data-driven attack detection trends in IoT research. The variations within the range of IoT-related datasets studied demonstrate that momentum is building in this area. However, the analysis provided indicates that there is still a need for the refinement of the development of such datasets in order to address their shortcomings with respect to feature engineering, IoT protocols, system requirements and efficiencies of detection models. Further, evolving trends, such as privacy-preserving techniques that employ the use of FL, demonstrate that IoT networks provide fertile ground for future experimentation in developing security solutions. Adapting to changing technology and understanding the IoT network better will help researchers and cybersecurity personnel in implementing robust solutions against attacks. However, it is important to remember that IoT sensors are usually limited in their functionalities, memory and computational capabilities. It is therefore necessary to provide solutions that are scalable and with little added overhead. For example, DL algorithms have the capability of selecting the best features and providing better detection results; however, they require more processing power compared to ML algorithms. Also, implementing FL on networks with simple IoT sensors (e.g., smart bulbs) may not be feasible, as bulbs do not have the resources to store and train local models. Hence, it is vital to keep all these considerations in mind while working towards better security solutions.

## Figures and Tables

**Table 2:** Feature comparison among IoT datasets.

Common Features	Bot-IoT	N-BaIoT	IoT NID	IoT-23	Med BIoT	MQTT-IoT	MQTTset	ToN_ IoT	Edge-IIoT	CICIoT 2023
Source IP address	✓		✓	✓		✓		✓	✓	
Destination IP address	✓		✓	✓		✓		✓	✓	
Source port	✓		✓	✓		✓		✓	✓	
Destination ports	✓		✓	✓		✓		✓	✓	
Transport-layer protocols	✓		✓	✓		✓		✓		✓
Timestamp	✓		✓	✓				✓	✓	✓
Total duration	✓		✓	✓				✓		✓
Source bytes	✓			✓				✓		
Destination bytes	✓			✓				✓		
Service				✓				✓		
Connection state				✓				✓		
Missed bytes				✓				✓		
Number of bytes per source IP				✓				✓		
Number of bytes per destination IP				✓				✓		
Number of packets per source IP				✓				✓		✓
Number of packets per destination IP				✓				✓		✓
MQTT message type						✓	✓	✓		
MQTT message length						✓	✓	✓		
User Name MQTT flag						✓	✓			
Password MQTT flag						✓	✓			
Will retain MQTT flag						✓	✓			
Will flag MQTT flag						✓	✓			
Clean MQTT flag						✓	✓			
Reserved MQTT flag						✓	✓			
All 100 of MedBIoT features		✓			✓					
Label/attack	✓			✓		✓		✓	✓	
Subcategory	✓									
Category	✓							✓	✓	✓

**Table 3:** Attack distribution in IoT datasets.

Dataset	Attack	A	N	T	D	M
**Bot-IoT**	Information gathering (service and OS scanning)		✓			
	TCP, UDP DoS/DDoS			✓		
	HTTP DoS/DDoS, information theft (keylogging, data theft)	✓				
**N-BaIoT**	BASHLITE/Mirai scan		✓			
	Mirai (ack flooding, syn flooding, UDP flooding, UDP plain flooding), BASHLITE (junk, UDP flooding, TCP flooding, COMBO attack)			✓		
	BASHLITE COMBO attack					✓
**IoTNID**	Scanning (host, port, OS)		✓			
	Man-in-the-middle	✓	✓			
	DoS attacks, Mirai (UDP, ACK)			✓		
	Mirai (HTTP flooding, brute force)	✓				
**IoT-23**	Mirai, Torii, Hide and Seek, Muhstik, Hakai, Internet Relay Chat Botnet (IRCBot), Hajime, Trojan, Kenjiro, Okiru, Gagfyt					✓
**MedBIoT**	Botnet malware: Mirai, BASHLITE and Torii					✓
**MQTT-IoT**	Aggressive scan		✓	✓		
	UDP scan			✓		
	Sparta Secure Shell (SSH) brute force, MQTT brute-force attack	✓				
**MQTTset**	Flooding denial of service,		✓	✓		
	MQTT Publish flood, Slow DoS against Internet of Things Environments (SlowITe), malformed data, brute-force authentication	✓				
**ToN_IoT**	scanning,		✓			
	DoS, DDoS, and man-in-the-middle attacks		✓	✓		
	Ransomware, backdoor, injection, cross-site scripting, password	✓				
**Edge-IIoT**	DoS/DDoS (ICMP), MiTM (DNS spoofing)		✓			
	MiTM (ARP spoofing),				✓	
	DoS/DDoS (TCP SYN, UDP)			✓		
	Information gathering (port scan, OS fingerprinting, vulnerability scan),		✓	✓		
	HTTP DoS/DDoS, injection attack (XSS, SQL injection, uploading attack), malware (backdoor, password cracking, ransomware)	✓				
**CICIoT2023**	ACK fragmentation, UDP flood, UDP plain flood, RSTFIN flood, PSHACK flood, TCP flood, SYN flood, synonymous IP flood			✓		
	ICMP flood, ICMP fragmentation, DNS spoofing, ping sweep, OS scan, vulnerability scan, port scan, host discovery, GREIP flood, Greeth flood		✓			
	SlowLoris, HTTP flood, SQL injection, command injection, backdoor malware, uploading attack, XSS, browser hijacking, dictionary brute-force	✓				
	ARP spoofing				✓	

**A:** application layer**, N:** network layer**, T:** transport layer, **D:** datalink layer, **M:** multiple layers.
